# Deep Learning Sensor Fusion for Autonomous Vehicle Perception and Localization: A Review

**DOI:** 10.3390/s20154220

**Published:** 2020-07-29

**Authors:** Jamil Fayyad, Mohammad A. Jaradat, Dominique Gruyer, Homayoun Najjaran

**Affiliations:** 1 School of Engineering, University of British Columbia, Kelowna, BC V1V 1V7, Canada; jfayyad@alumni.ubc.ca; 2 Department of Mechanical Engineering, American University of Sharjah, Sharjah, UAE; mjaradat@aus.edu; 3 Department of Mechanical Engineering, Jordan University of Science & Technology, Irbid 22110, Jordan; 4 PICS-L, COSYS, University Gustave Eiffel, IFSTTAR, 25 allée des Marronniers, 78000 Versailles, France; dominique.gruyer@univ-eiffel.fr

**Keywords:** autonomous vehicles, self-driving cars, deep learning, sensor fusion, perception, localization and mapping

## Abstract

Autonomous vehicles (AV) are expected to improve, reshape, and revolutionize the future of ground transportation. It is anticipated that ordinary vehicles will one day be replaced with smart vehicles that are able to make decisions and perform driving tasks on their own. In order to achieve this objective, self-driving vehicles are equipped with sensors that are used to sense and perceive both their surroundings and the faraway environment, using further advances in communication technologies, such as 5G. In the meantime, local perception, as with human beings, will continue to be an effective means for controlling the vehicle at short range. In the other hand, extended perception allows for anticipation of distant events and produces smarter behavior to guide the vehicle to its destination while respecting a set of criteria (safety, energy management, traffic optimization, comfort). In spite of the remarkable advancements of sensor technologies in terms of their effectiveness and applicability for AV systems in recent years, sensors can still fail because of noise, ambient conditions, or manufacturing defects, among other factors; hence, it is not advisable to rely on a single sensor for any of the autonomous driving tasks. The practical solution is to incorporate multiple competitive and complementary sensors that work synergistically to overcome their individual shortcomings. This article provides a comprehensive review of the state-of-the-art methods utilized to improve the performance of AV systems in short-range or local vehicle environments. Specifically, it focuses on recent studies that use deep learning sensor fusion algorithms for perception, localization, and mapping. The article concludes by highlighting some of the current trends and possible future research directions.

## 1. Introduction

Autonomous vehicles (AVs) have made impressive technological progress in recent years; these noticeable advancements have brought the concept of self-driving cars into reality. According to a report published by the U.S. Department of Transportation, 94% of vehicle crashes occur due to driver behavior [1]. For this reason, AVs are projected to lower the risk of drastic accidents and increase road safety. Additionally, it is anticipated that AVs will assist in reducing carbon emission levels, and hence protect the environment [2]. Moreover, self-driving cars are expected to smoothen traffic flow, increase productivity, and have enormous economic impacts.

**Figure 1:**
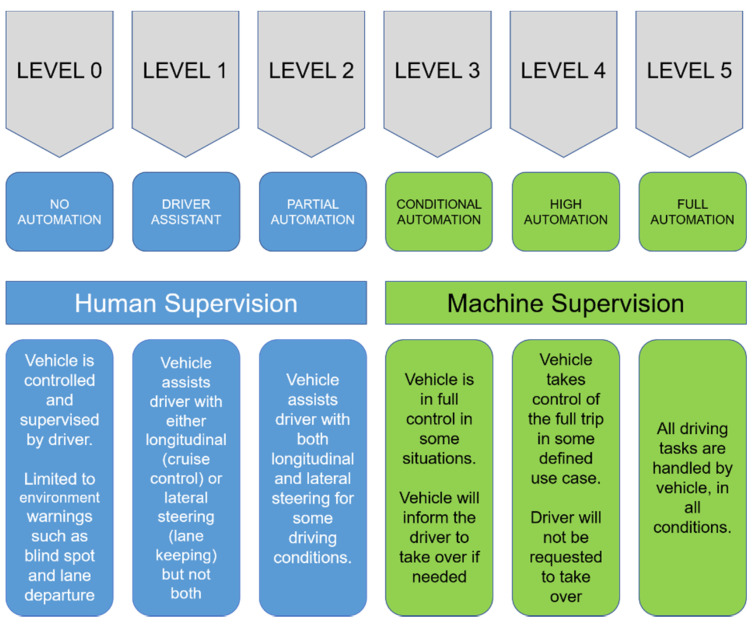
The six levels of autonomous vehicles as described by the Society of Automobile Engineers (SAE) [3], their definitions, and the features in each level.

According to the Society of Automobile Engineers (SAE) [3], there are six different levels of automated vehicles, starting from level 0 where the driver is in full control of the vehicle, and ending with level 5 where the vehicle is in full control of all driving aspects. These levels are portrayed in Figure 1**.** Currently, it can be confidently stated that levels 2 and 3 are being adopted in some of the commercial cars, such as GM’s Cruise [4], Tesla’s Autopilot [5], and BMW [6]. Several autonomous features are already being performed in these cars, such as adaptive cruise control, automatic braking, and lane-keeping aid systems.

**Figure 2:**
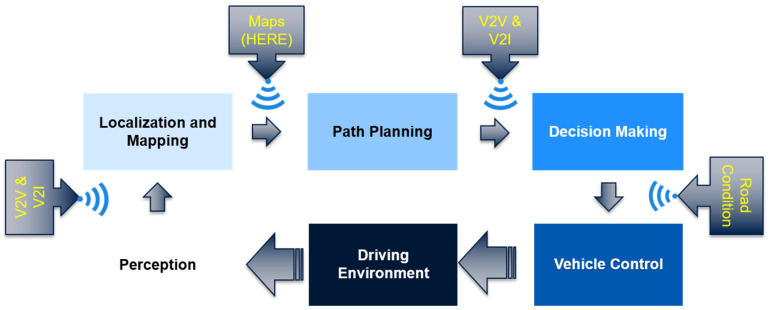
Full autonomous navigation system. Sensor technology and sensor fusion overview. V2V, vehicle-to-vehicle; V2I, vehicle-to-infrastructure.

Although different AV systems may differ slightly from one to another, they all need to present a solution for the autonomous navigation problem, which is generally divided into four main elements: perception, localization and mapping, path planning, and control. In perception, the vehicle utilizes a group of onboard sensors to detect, understand, and interpret the surrounding environment, including static and dynamic obstacles, such as other moving vehicles, pedestrians, road signs, traffic signals, and road curbs. Localization and mapping tasks attempt to locate the vehicle globally with respect to world coordinates. Additionally, they are responsible for building a map of the vehicle’s surroundings and continuously tracking the vehicle’s location with respect to that map. Subsequently, path planning exploits the output of the previous two tasks in order to adopt the optimal and safest feasible route for the AV to reach its destination, while considering all other possible obstacles on the road [7]. Lastly, based on the selected path, the control element outputs the necessary values of acceleration, torque, and steering angle for the vehicle to follow that selected path [8]. Additionally, multiple studies consider adding connected vehicle technologies [9,10], such as vehicle-to-vehicle (V2V) and vehicle-to-infrastructure (V2I) technologies, where essential information is shared to create an enhanced cooperative driving environment, as shown in Figure 2. This extended and improved cooperative perception allows vehicles to predict the behavior of the key environmental components (obstacles, roads, ego-vehicles, environment, driver behavior) efficiently and to anticipate any possible hazardous events.

One of the major considerations in any AV system is the selection of the proper group of sensors and their optimal configuration, which will be utilized to mimic the human ability to sense and create a reliable picture of the surroundings. It is always important to take into consideration the advantages, disadvantages, and limitations of this group of sensors as a whole, i.e., a logical and smart sensor. In many cases, the overall performance of the system is substantially improved when multiple sensors operating on different wavebands are placed to collaborate and produce a fused output. Consequently, sensor fusion is a vital process that is required in all AV systems to overcome the limitations of individual sensors, and hence improve the efficiency of the overall AV system.

Presently, there is an enormous amount of effort invested in improving the performance, reliability, robustness, and accuracy of self-driving vehicles modules, not to mention the cybersecurity and safety operating issues that can also be critically important under real driving conditions. While keeping in mind that vehicles are present in an environment that is highly complex, fast, and dynamic, the applied algorithms should be crafted in a special way that balances accuracy and fast real-time processing. With the emergence of new powerful computational technologies, such as graphics processing units (GPUs) and the availability of a large amount of data (so-called “big data”), a subset of artificial intelligent (AI) and machine learning known as deep learning has gained huge popularity in several applications related to object detection, object identification, road situation recognition, and more generally robotics issues [11]. Deep learning algorithms have been utilized in different aspects of AV systems, such as perception, mapping, and decision making. These algorithms have proven their ability to solve many of these difficulties, including computational loads faced by traditional algorithms while maintaining decent accuracy and fast processing speed.

This review paper will focus on two components of the AV systems: perception, and localization and mapping. The main aim is to provide a comprehensive review of the most useful deep learning algorithms in the field of sensor fusion for AV systems. The paper is organized as follows. Section 2 provides an overview of the advantages of recent sensor combinations and their applications in AVs, as well as different sensor fusion algorithms utilized in the field. Section 3 describes the task of environmental perception and provides an overview of the latest deep learning detection algorithms, along with an analysis of their performance. Section 4 discusses approaches for localization and mapping, and compares different sensors used for that task. Additionally, it evaluates various applications of deep learning algorithms and compares them with the traditional algorithms. Section 5 provides future research recommendations that might bridge the gap in research topics related to AV systems.

## 2. Sensor Technology and Sensor Fusion Overview

Sensors are generally categorized into two classes based on their operational principle. Proprioceptive sensors are the first category in which the sensor operates by capturing the dynamical state and the internal measurements of the dynamic system. Global positioning system (GPS), encoders, gyroscopes, gyrometers, and accelerometers are examples of this category. The second class covers exteroceptive sensors, with which the external variables of the surrounding system are sensed. This applies to cameras (Complementary Metal-Oxide-Semiconductor (CMOS), Infrared, fisheye, and cyclops), radio detection and ranging (radar), and light detection and ranging (LiDAR). In addition to this categorization, sensors can be either passive or active. Passive sensors produce outputs by sensing the surrounding energy using cameras and GPS, while active sensors transmit energy and measure the reflected energy to produce outputs (LiDAR and Radar) [12].

In autonomous vehicles, different combinations of active and passive sensors are employed to perform the two main tasks of perception. (1) Environmental Perception: RGB cameras, thermal cameras, LiDAR, and radar are used for on-road vehicle detection and tracking, pedestrian detection, tracking, road surface detection, road lane detection, and road sign detection. (2) Localization: global navigation satellite systems (GNSS), inertial measurement units (IMU), inertial navigation systems (INS), odometers, cameras, and LiDAR are used to obtain the relative and absolute positions of the vehicle.

In general, it is difficult to generate data from a single independent source and use it in complex applications, such as AVs. The reasons are either due to sensor shortages, the nature of the sensed environment, or both. Sensors suffer from several inadequacies and limitations, which can degrade their performance. Some sources of performance degradation are due to drifting errors, where a small offset can lead to a huge error when readings are accumulated over time, as in IMU [13,14]. Additionally, errors can be due to low sensor resolution, surface irregularities, or wheel slipping, as in-wheel odometers. Finally, they can be due to uncertainty in readings. Some high-accuracy sensors exist that could overcome some of these limitations, such as differential global positioning systems (DGPS), real-time kinematic (RTK) positioning systems, and fiber optics IMU; however, they are often unavailable or impractical for use in AV systems due to their operating limits (occultation and multireflection effect) and their high cost.

Besides the sensors’ own imperfections, the sensed environment conditions have an enormous effect on the sensors’ outputs. Sensor noise, for example, disturbs camera images through sunlight intensity and illumination. Similarly, low light at nighttime degrades the outputs of color cameras. Moreover, GPS sensors are affected by outages in certain areas, such as tunnels and forests.

AV researchers use different combinations of sensors and fuse their readings at different levels in order to compensate for the limitations of the individual sensors. Vision cameras are essential sensors that generate a detailed environmental view of the AV surroundings. They are inexpensive sensors for a given level of performance (e.g., resolution, accuracy) compared to active ranging sensors, and can provide dense pixel information for the surrounding scene at relatively low cost. However, normal vision-based systems fail to provide the depth information needed to model the 3D environment. One alternative is to use a stereovision system that consists of multiple cameras with different locations. Nevertheless, these systems are also extremely sensitive to external environmental conditions, such as light intensity (low light and direct sunlight) [15]; and severe weather situations such as fog [16,17], snow, and rain. Fusing a vision-based system with LiDAR, for instance, creates a complementary output that provides the depth information while being robust to external weather conditions [18,19,20].

The use of infrared and thermal imaging is another active field that researchers often investigate for environment perception applications, especially in unfavorable light conditions and night vision. These systems are often used for applications such as pedestrian detection and tracking [21,22,23] due to their ability to detect humans regardless of the light intensity. In the literature, thermal cameras have been fused with either RGB-D [24] or LiDAR sensors [25,26] to add depth, and hence improve the system performance; however, this advantage can be dramatically compromised in extreme weather conditions, such as high temperatures.

Localization and mapping normally use a combination of different sensors, such as GPS, IMU, LiDAR, and cameras, to obtain accurate and reliable results. Despite the availability of highly accurate and reliable GPS sensors, it is common that GPS signals typically face blockages or outages in certain environmental conditions. Hence, to compensate for losses of GPS signal, the localization system is likely to be coupled with other sensors, such as IMUs [27].

Additionally, high-accuracy sensing devices are usually very expensive, making them unsuitable for use in applications other than accurate ground truth readings for evaluation and validation of the quality and the performance of an algorithm. Reducing the cost of sensing technologies while maintaining an efficient output is one of the priorities in AV systems, hence combining low-cost IMU data and GPS signals can yield continuous and accurate state estimations of vehicles [28]. Moreover, cameras and LiDAR [29,30] are used in a configuration that will allow extraction of specific environment primitives (road markings and static interest points) for use in either map building through simultaneous localization and mapping algorithms (SLAM) [31] or by matching them with a pre-existing high-definition (HD) map [32] and then obtaining accurate positions for both the ego vehicle and surrounding objects.

Table 1 provides a comprehensive list of various combinations, fusions, and association methods of the most common sensors used in self-driving vehicles. The table also describes the limitations of the sensors if they are to be used individually. Additionally, it lists the advantages of fusing a suitable set of sensors to achieve the desired output.

Both sensor fusion and information fusion can be defined as the process of managing and handling data and information coming from several types of sources in order to improve some specific criteria and data aspects for decision tasks. In our case, the process of fusion consists of combining the outputs of individual sensors or the outputs of specific algorithms (state vectors and uncertainty matrices) to produce a new combined outcome that is enhanced, extended, enriched, more reliable, more confident, and more robust than those generated by each of the individual sensors separately. The final goal is to use the redundancies, complementarities, and advantages of a set of data in order to obtain good enough perception data to make the best decision.

**Figure 3:**
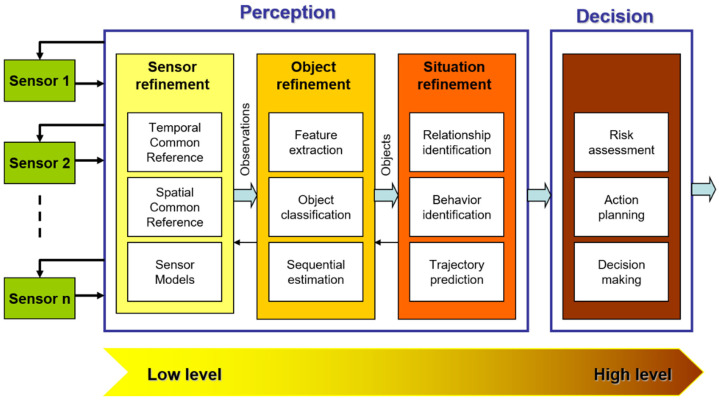
The different stages in the perception and the decision process.

Figure 3 illustrates the five different levels of data processing for perception and decision applications. The first level represents the raw input data collected from various combinations of sensors. The second level portrays the process of filtering, spatial and temporal alignments, and uncertainty modeling. The outputs of the latter are observations, which will in turn pass to the third level, where feature extraction, object detection, clustering, data processing occur to generate representations of objects (e.g., sizes, shapes, colors). Layer four concludes the perception layer, where different objects can be identified by their behavior or specific properties and trajectories to build a proper representation of their interactions, which are inputs to higher-level processing, such as decision making at the fifth level. It is worth mentioning that an output is either used for one of the perception levels (tracking stage, information looping, or strong coupling) or used for the final decision layer [33].

The field of sensor fusion has been applied in multiple applications, ranging from military applications such as automated target recognition [56] to medical applications [57], remote sensing [58], and self-driving vehicles [59]. In autonomous vehicles, sensing of the surrounding environment is one of the crucial steps in building a successful and complete system (perception, decision, and action). Vehicles are usually equipped with different types of sensors that collaborate in order to initiate the right decisions. With that said, an enormous amount of research has been conducted over the past decade to adopt and improve sensor fusion methods for autonomous vehicles.

**Figure 4:**
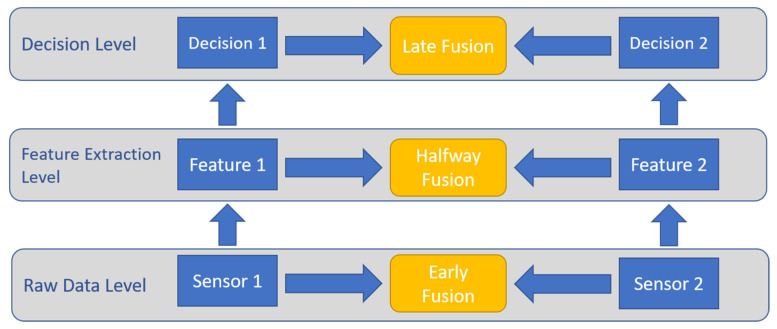
Sensor fusion architecture described in terms of the three different levels. Level one represents early fusion, level two represents halfway fusion, and level three represents late fusion.

Searching the literature, it has been found that several categorization schemes of sensor fusion methods exist. In this section, the most used classes will be listed. The first category is based on the input type that is used in the fusion network, which includes data fusion (early fusion), where the fusion takes place at the raw data level. The second category is feature fusion (halfway fusion), where features are first extracted from sensor data and then these features are fused halfway through the network. The last category is decision fusion (late fusion), in which multiple classifiers are used to generate decisions that are then combined to form a final decision. The architecture of the different levels is illustrated in Figure 4.

A different categorization was explained by Dasarathy in [60], where he listed five detailed classification classes, including:

1. **Data in, data out**: The input to the fusion network is raw sensor data, while the output is processed (typically enhanced) raw data.
2. **Data in, feature out**: Raw data is integrated to produce a set of output features.
3. **Feature in, feature out**: Where the input and output of the fusion network are feature vectors. This class is commonly referred to as either feature fusion, symbolic fusion, or information fusion.
4. **Feature in, decision out**: As the name suggests, the input is a feature vector and the output is a decision. This class is more common in pattern recognition activities, where feature vectors are processed to be labeled.
5. **Decision in, decision out**: Where both inputs and outputs are decisions, usually referred to as decision fusion networks.

Based on the source of the fused data, sensor fusion can also be categorized as a multimodal fusion method [61], where the fused data are obtained from two or more different types of sensors. Fusing LiDAR point cloud and camera images is a good example of this type of fusion, where the two modalities complement the functionality of each other and provide an improved outcome. Another class of this category is multitemporal fusion, where data are obtained from the same sensor but at different acquisition times. This type of fusion is common in satellite images used for monitoring changes on Earth. The third type of fusion is multifocus fusion [62], where images are obtained from different focal lengths. The last class is multispectral fusion [63], in which images are captured from different wavelength sensors, such as an RGB camera and a thermal camera. This type of fusion is found in applications similar to pedestrian detection and object recognition.

Additionally, based on sensor configuration, sensor fusion can be categorized into a complementary configuration, in which two independent sensors are used and their outputs are combined to complement each other. A perfect example of this type is the fusion of multiple ultrasonic sensors fixed on a robot bumper to expand the coverage area. Fusion can also be of a competitive configuration (also called redundant configuration), where multiple sensors are used to measure the same property and the outputs are used for correction purposes, as is the case in multiple IMU measurements. The last configuration is the cooperative configuration, where two or more sensors are used to provide an output that cannot be achieved by the individual sensors, such as integrating the outputs of two stereovision cameras in order to get a three-dimensional depth image.

Lastly, based on fusion architecture, sensor fusion can be categorized into centralized, decentralized, and hybrid fusion architectures [33]. In centralized fusion, all data from the different sensors are connected to a central processing unit. After all data are aligned to a common reference frame, the central unit receives the output as one source of information in order to fuse it. In decentralized fusion, data obtained from sensors are processed locally, then the obtained output is forwarded to a global processing unit for fusion. A hybrid architecture includes sets of data processed locally and forwarded to the global processor, where the remaining data will be processed and fused.

This review paper divides sensor fusion techniques and algorithms into classical algorithms and deep-learning-based algorithms. However, the scope of this study is to review the implementation of deep learning sensor fusion approaches in AV applications.

### 2.1. Traditional Sensor Fusion Approaches

**Figure 5:**
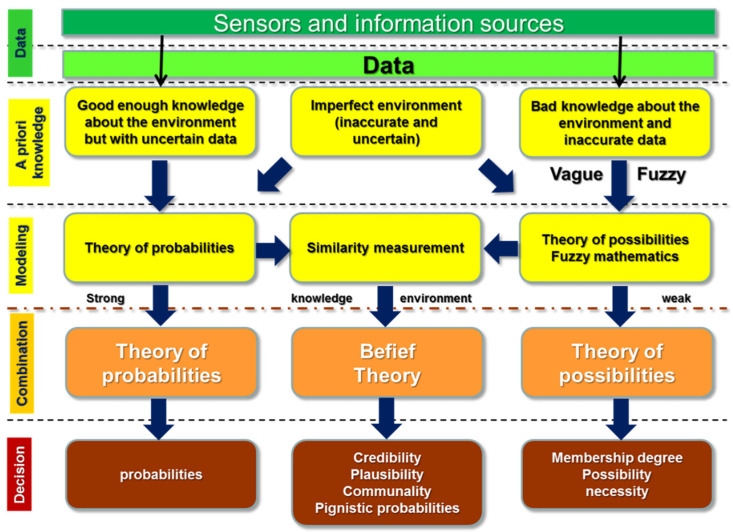
Theories of uncertainty for modeling and processing of “imperfect” data.

**Figure 6:**
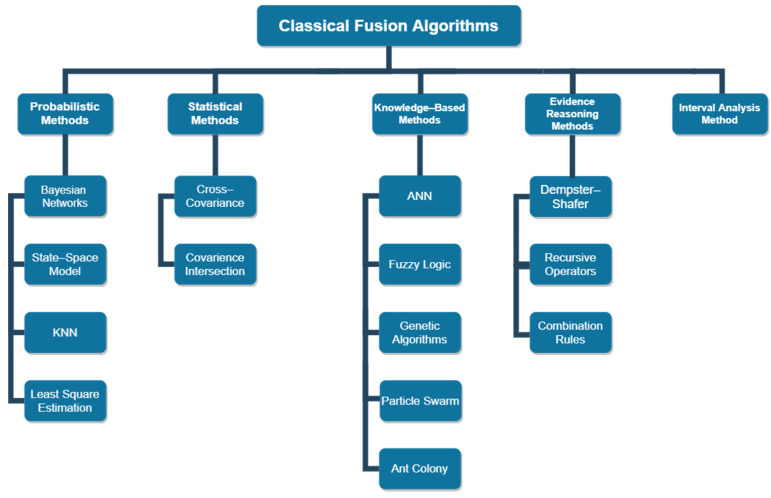
Classical approaches for sensor fusion algorithms.

There are several classical algorithms that utilize data fusion for the development of applications that require to modeling and propagation of data imperfections (inaccuracy, uncertainty). These algorithms apply methods and approaches that are based on theories of uncertainty, as illustrated in Figure 5 These methods include probabilistic methods, statistical methods, knowledge-based methods (fuzzy logic and possibility), interval analysis methods, and evidential reasoning methods. The variations of each category are listed in Figure 6 In this section, Table 2 briefly summarizes the common classical algorithms, along with their advantages and disadvantages. The readers interested in detailed discussions about traditional fusion algorithms are recommended to refer to [33,64,65,66].

### 2.2. Deep Learning Sensor Fusion Approach

Deep learning could be seen as an improvement of neural networks and is a subdivision of artificial intelligence and machine learning that aims to imitates the functionality of the human brain. The algorithms involve creating manifold networks that have multiple layers, allowing them to process raw data and extract certain patterns to perform complex and intelligent tasks. The core concept of deep learning is based on artificial neural networks (ANN), which can be traced back to 1943, when Walter Pitts and Warren McCulloch [76] took the first steps towards building a model that based on the working principle of a human’s brain neural networks. While the basics of deep learning were founded long ago, its recent vast emergence is due to the development of powerful computing machines and the availability of the “big data” needed to train the models. Recently, deep learning is being extensively used in many different applications, such as in object detection [77], environment segmentation, semantic object identification, healthcare [78], self-driving vehicles [79,80], and finance [81], to name a few.

Several different algorithms exist that are listed under the category of deep learning. Each technique has its own unique properties, and hence is used for a certain application, where the goal is to achieve optimal performance. The frequently used deep learning methods can be listed as (1) convolutional neural networks (CNN), (2) recurrent neural networks (RNN), (3) deep belief networks (DBN), and (4) autoencoders (AE). Table 3 outlines an overview of these algorithms, along with their applications.

**Figure 7:**
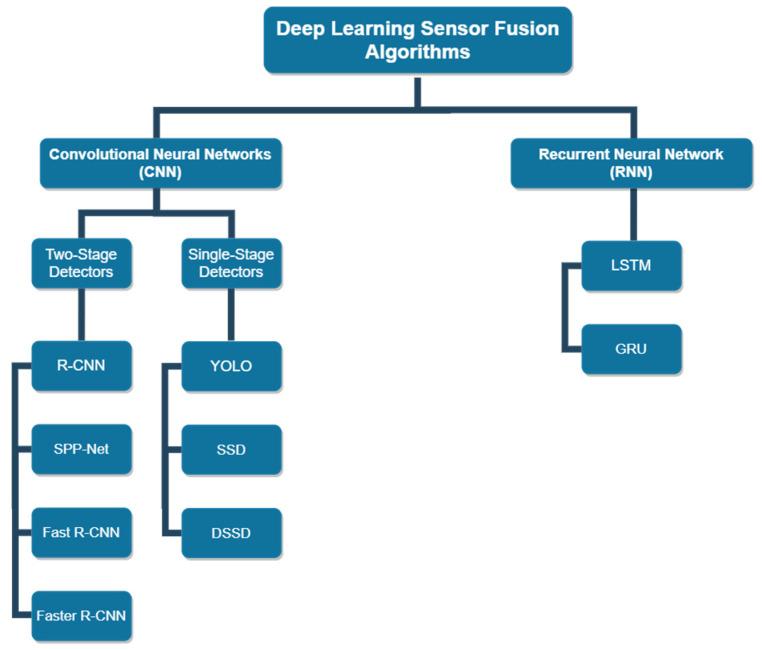
Common deep learning sensor fusion algorithms used in autonomous vehicle applications. R-CNN: Region-Based CNN; SPP-Net: Spatial Pyramid Pooling network; YOLO: You only look once; SSD: Single-Shot Multibox Detector; DSSD: Deconvolutional Single-Shot Multibox Detector; LSTM: Long-Short Term Memory; GRU: Gated Recurrent Unit.

There is a noticeable increase in the amount of research associated with deep learning sensor fusion algorithms in autonomous driving. CNN and RNN are among the most commonly used algorithms in AVs. This paper, hence, aims to provide a detailed overview of the recent advancements in sensor fusion using deep learning approaches, while focusing on these two algorithms and their variations. Figure 7 depicts different variations of CNN and RNN that have been utilized in AV applications.

In this review, the discussions around the power of deep learning methods are concentrated on:

1. Environmental perception, including vehicle detection, pedestrian detection, road surface detection, lane tracking, and road sign detection.
2. Localization and mapping.

## 3. Environmental Perception: Local Dynamic Perception Map

Environmental perception is the process by which AVs tend to sense, understand, and build a full awareness of the environment and the objects that surround it. This perception map is built from the information coming from the five main key components of the environment (obstacle, road, ego vehicle, environment, and driver). Vehicles are usually equipped with multiple sensors to assess the first two key components and detect a variety of objects in the environment, such as vehicles, pedestrians, signs, roads, and lanes. Generally speaking, RADAR, LiDAR, and vision-based systems are the most common sensors used in environmental perception. Therefore, it is very common to find literature discussing detection algorithms using convolutional neural networks (CNN), as they are extremely powerful with visual data.

Before CNN was introduced in 2012, multilayer perceptron (MLP) was heavily used in image recognition and classification. MLP is a feed-forward, fully connected neural network, which consists of an input layer, a hidden layer, and an output layer. With current advancements, it has been concluded that MLP has many limitations and is not a sufficient tool due to the following disadvantages. First, it has a growing number of parameters that need to be trained. Second, it loses spatial information and pixel arrangement of an image. Third, it is not translation-invariant. On the other hand, CNN is a subset of deep learning algorithms that uses convolution operation to process pixels in images. It has a different architecture compared to that of a MLP; the layers are organized into three dimensions of width, height, and depth. Additionally, the neurons of CNN are not fully connected to the layers.

**Figure 8:**
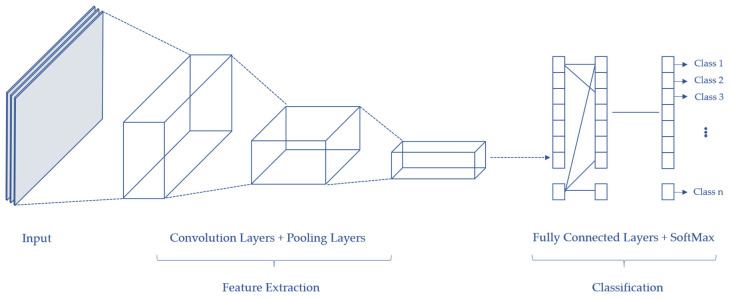
The different processing layers in a convolutional neural network (CNN) for object detection and identification.

A general CNN usually consists of the following layers, as shown in Figure 8:

- Input layer: This contains the data of the input image.
- Convolution layers: Convolution operation is performed in this layer to extract important features from the image.
- Pooling layers: Located between two convolution layers, which help in minimizing the computational cost by reducing some—but maintaining the most dominant—spatial information of the convoluted image.
- Fully connected layer: This serves as a classifier connecting all weights and neurons.
- Output layer: This stores the final output, which is the classification probability.

**Figure 9:**
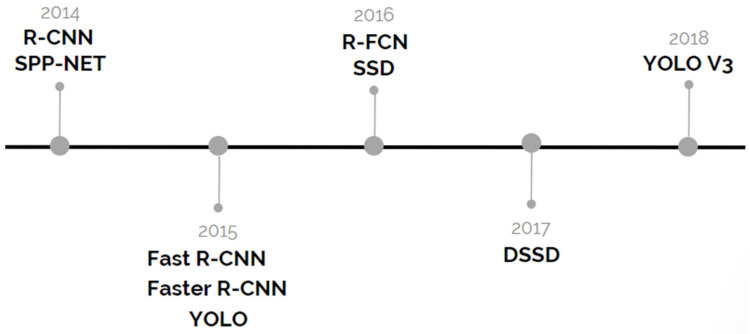
Timeline development of CNN-based detectors. R-FCN: Region-Based Fully Connected Convolution Network.

CNN achieved its current popularity in 2012 [95] when Krizhevsky et al. proposed AlexNet and won the ImageNet Large-Scale Visual Recognition Challenge (ILSVRC). Since that breakthrough, there has been increased research interest in CNN. Going down the timeline, advancements in CNN image detectors have gone through two parallel paths: (i) the two-stage detectors, which consist of region proposals first, then prediction; and (ii) the single-stage detectors, in which prediction is carried out directly without having an intermediate stage. Figure 9 presents the timeline of the most popular CNN detectors of both types.

### 3.1. R-CNN

**Figure 10:**
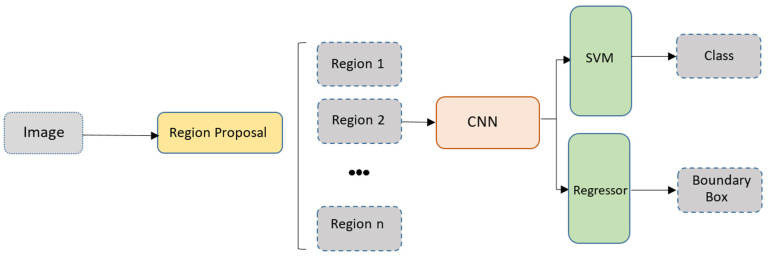
The architecture of the region-based CNN (R-CNN) algorithm.

Region-based CNN (R-CNN) was the first two-stage detector introduced by Girshick et al. [96]. The purpose of this algorithm is to reduce the computation load and enhance the detection speed. This was achieved by creating 2000 regions in the image through a selective search algorithm instead of covering all regions of the image. Selected regions are processed by a CNN network for feature extraction, and later classified by a Support Vector Machine (SVM) classifier. The architecture of R-CNN is illustrated in Figure 10. Sensor fusion based on R-CNN is applied to different applications of AVs. Wanger et al. [39] studied the impact of fusing thermal images and visible images in pedestrian detection in both daytime and nighttime. In their research, the R-CNN algorithm was used with both early fusion (pixel-level) and late fusion architectures. Their results showed that pretrained late fusion architecture achieved better performance compared to state-of-the-art baseline algorithms. Similarly, in a different study, LiDAR depth features known as horizontal disparity, height above ground, and angle (HHA) features were fused with RGB images to detect pedestrians [34]. A R-CNN network was used and six different fusion architectures were generated based on the network layer at which the fusion takes place. While both studies showed improvements in the mean percentage error, the proposed approach still requires more work to reduce the processing time in order to be fully efficient in embedded real-time AV applications.

### 3.2. SPP-Net

**Figure 11:**
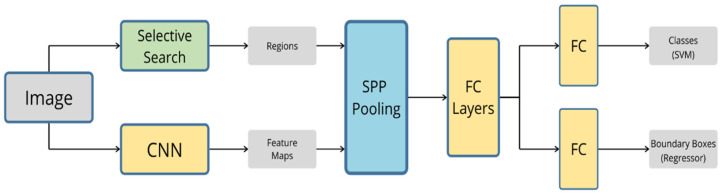
The architecture of spatial pyramid pooling (SPP-Net) algorithm.

Despite the astonishing accuracy of R-CNN at the time, it was not optimal enough to be used in real-time autonomous driving application. The algorithm requires around 47 s for image detection, which is too long for real-time applications. In addition, R-CNN has to classify around 2000 proposed regions, which leads to massive training time. Multiple attempts have been made to overcome these drawbacks. One of these attempts was the introduction of spatial pyramid pooling (SPP-Net) [97]. This new technique has the advantage of eliminating the need for cropping, resizing, or changing the aspect ratio of the input image to a certain size by introducing multiple pooling layers with different scales. In addition to its ability to generate a fixed-length representation regardless of the input size, SPP-Net processes the full image at once instead of processing all 2000 regions generated by the region proposal, which leads to a noticeable improvement in the processing speed of the algorithm. The simplified architecture of the algorithm is illustrated in Figure 11, where regions and feature maps are passed into multiple pooling layers, before they are concatenated and fed into a Fully Connected (FC) Layer for classification and regression.

### 3.3. Fast R-CNN

**Figure 12:**
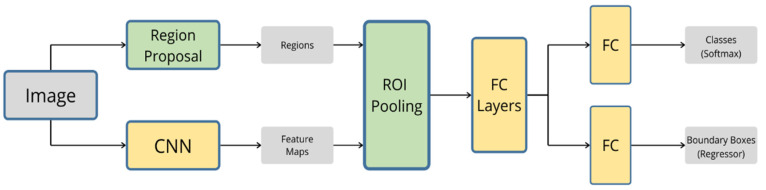
The architecture of fast R-CNN.

Fast R-CNN was also proposed by Girshick [98] to improve the speed of training and testing and to improve detection accuracy. In fast R-CNN, instead of processing the region proposals by the CNN network, the input image is processed and a convolutional feature map is produced. Regions of interest (ROI) are generated from the feature maps and fed to the fully connected layer, as shown in Figure 12. It is worth mentioning that fast R-CNN is nine times faster in training and 213 times faster in inferencing than R-CNN [98].

### 3.4. Faster R-CNN

**Figure 13:**
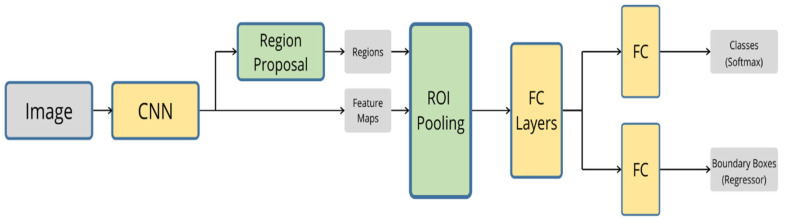
The architecture of faster R-CNN.

Even though inferencing time was decreased from 47 s in R-CNN to 2.3 s in fast R-CNN, the latter algorithm determines the regions and their bounding boxes using a selective search algorithm, which itself causes a considerable delay in the process. In 2015, Ren et al. proposed the region proposal network (RPN), which is a separate neural network used to predict the bounding boxes. This network is merged with R-CNN, which share the convolution features. The new algorithm, named faster R-CNN [99], has the architecture described in Figure 13. Faster R-CNN was the first place winner of the ILSVRC competition. Faster R-CNN reports a testing time of 0.2 s, which makes it suitable for real-time applications.

In comparison, faster R-CNN is noted as being the most used among other region-based CNN algorithms due to its accuracy and fast processing time. Liu et al. [37] used faster R-CNN in multispectral pedestrian detection, where thermal and color images are fused to provide the complementary information required for daytime and nighttime detection. Having complementary sensor data would undoubtedly enhance the detection results; however, choosing the correct fusion architecture would yield a better detection outcome. In [37], four fusion models named early fusion, halfway fusion, late fusion, and score fusion were designed and tested. It was found that halfway fusion achieved the best detection results compared to those of the baseline faster R-CNN method. By extending the work done in [37], two additional fusion architectures were added in [100], namely the input fusion and score fusion II. Additionally, an illumination-aware gating network that assigns different weights to the modalities based on the illumination condition was added. In a different approach, faster R-CNN was used in [101] to detect pedestrians at nighttime by fusing successive images from a monochrome image. It is claimed that successive frames can improve the detection results by increasing the information contained in the image, especially in dark conditions with low brightness and contrast.

Table 4 provides a quantitative comparison of the two-stage detector algorithms. It compares the time needed to train each of the networks with respect to the baseline algorithm (R-CNN). Additionally, the table lists the rate at which each algorithm needs to perform image recognition. The data shown in the table are from the experimental results reported in each corresponding study [96,97,98,99].

### 3.5. YOLO

**Figure 14:**
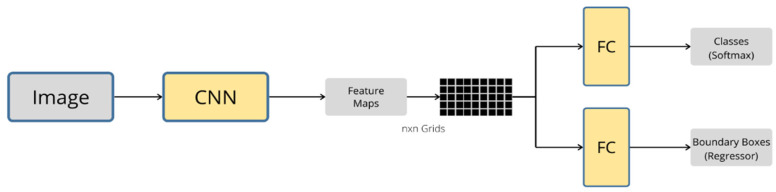
The architecture of “you only look once” (YOLO) algorithm.

Single-stage detectors, on the other hand, consist of a one-step regression rather than a multistage classification process. One of the most popular algorithms is the “you only look once” detector (YOLO), founded in 2016 by Redmon et al. [102]. As seen in Figure 14, the input image is divided into a defined number of grids, then a single neural network is applied to predict bounding boxes and produce class probability for the boxes, which is all performed in one stage. Compared to the previous detectors mentioned above, YOLO is considered to have a very fast detection speed of 45 frames per second [102]; however, the use of YOLO is restricted due to its disadvantages of high localization error and low detection accuracy when dealing with small objects. These limitations were addressed by proposing improved algorithms in YOLOv2, YOLO9000 [103], and YOLOv3 [104].

In many research attempts, LiDAR and visible cameras were used together to obtain better detection results. In [105], for example, Asvadi et al. used depth maps (DMs), while reflectance maps (RMs) generated by 3D LiDAR were fused with RGB images to detect objects on the road. Three YOLO networks were used to process DM, RM, and RGB images separately and generate bounding boxes from each network. Features are extracted from the three modalities and a decision-level fusion is then applied to achieve vehicle detection. In a different study [106], LiDAR point cloud data were used to construct a map of the vehicle’s view; then, these maps were used to generate regions of interest, which were then projected on the camera image. A YOLOv3 network was then utilized to perform real-time vehicle detection. In spite of the decent results that were presented in both papers, small objects such as pedestrians were not considered.

### 3.6. SSD

Throughout the literature, it has been noticed that the YOLO algorithm is mostly applied on large objects such as vehicles. In fact, according to [107], YOLO’s accuracy is degraded when dealing with small and variant-scale objects. Moreover, YOLO applies spatial constraints on the bounding boxes, limiting the classification to a single class of objects [108]. Many noticeable efforts have been put forth to solve such restrictions. One of the starting points is the single-shot multibox detector (SSD) [109], which is the result of a recent study involving significant improvements and attempts to overcome the limitations of the previous state-of-the-art methods. SSD is designed to have bounding boxes with different sizes and aspect ratios. This property enables the algorithm to detect different objects with different sizes in the same image. SSD is reported to be faster and more accurate than YOLO. It matches the accuracy of faster R-CNN, but with a speed of 59 frames per second (more than 2500 times faster).

**Figure 15:**
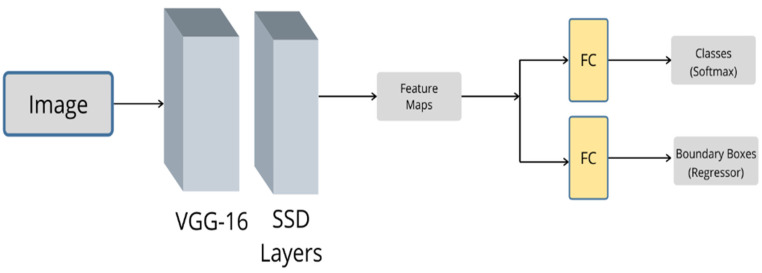
The architecture of the SSD algorithm. The CNN Network is VGG-16.

In [110], Kim et al. used the SSD algorithm for general object detection in the autonomous driving applications. LiDAR 3D point clouds were converted into 2D images, then these images were used along with RGB images as inputs for two separate SSD networks. Finally, gated fusion units (GFU) were used to assign selective weights to fuse both feature maps produced by the two SSD networks through a feature fusion level. The experimental results showed that the proposed GFU–SSD method outperformed the baseline SSD. The authors in [38] attempted to compare different fusion techniques with different CNN architectures while keeping SSD as the baseline detector. The fusion of thermal images and visible images was carried out with early and late fusion by using a SSD network and comparing it with other detectors, such as faster R-CNN and DefineNet. The results showed that the miss rate was reduced with the SSD detectors in both early and late fusion. Figure 15 illustrates the architecture of the SSD.

### 3.7. DSSD

Due to the fact that small objects yield a limited number of pixels and information, the detection of these objects becomes a burden. In most cases, the improvement of accuracy is traded with the speed of detection [111]. Some variations of the SSD networks have been implemented to improve the accuracy with small objects while maintaining high detection speed. For example, the deconvolutional single-shot detector (DSSD) [112] uses ResNet101 instead of the original Visual Geometry Group (VGG) classifier and adds more context information into the existing SSD algorithm by augmenting it with deconvolutional layers. This provides feature maps with better resolution, which enhances the detection of small objects. For the pedestrian detection task, colored and thermal images were fused using halfway fusion through the DSSD network [40]. As pedestrians are small objects, the new algorithm was compared to previous studies that use different detectors. It has been shown that the overall accuracy is improved.

## 4. Ego-Localization and Mapping

An effective autonomous driving system requires the vehicle to determine its position and orientation accurately. Vehicles need to have accurate, reliable, and robust localization algorithms to assist in their maneuvering tasks, avoid surrounding obstacles, and perform the right driving actions. Moreover, the localization system needs to be robust to handle variant complex environments and severe weather conditions. Generally, localization is commonly performed using a variety of sensors, such as GNSS; and dead reckoning devices, such as IMU, vision sensors, and LiDAR (for visual odometry, primitive detection, and mapping, for example for SLAM algorithms). The fusion of two or more of these sensors is also a common practice to enhance the overall localization performance.

It is worth mentioning that some emerging studies [113,114,115] propose different driving algorithms that avoid the need for localization and mapping stages, and instead sense the environment and directly produce end-to-end driving decisions. This is known as the behavior reflex approach [113]. In contrast to the classical method above, known as the “mediated perception approach”, this approach aims to reduce the computational load by eliminating unessential information, hence improving the speed of the process [113].

This section aims to analyze the localization techniques as part of the mediated perception approach, while focusing on the fusion of different sensors through deep learning algorithms. Table 5 provides a summary of the common ego-localization and mapping techniques.

### 4.1. GNSS/IMU-Based Localization

GNSS is one category of the most commonly used sensors for localization in autonomous vehicles. They have the advantages of low production cost and ease of integration within the vehicle system. GNSS technology, however, has two main deficiencies that prevent them from being a reliable standalone source of information. The first disadvantage is their insufficient accuracy (in the range of 10 m). This range is unsatisfactory for AV applications, where accuracy in the centimeter range is required. The second disadvantage is signal blockage and multipaths, where GNSS signals can sometimes be interrupted in real driving environments. An adequate amount of research exists that focuses on solving these two shortages; some of it has already provided acceptable results, which are discussed below.

DGPS and RTK-GPS are used to enhance the accuracy of GNSS. Both DGPS and RTK rely on having a base GPS station that has a known position of high accuracy. In the case of DGPS, the base station uses its position for comparison with that calculated by GPS and sends the difference to the receivers in order to use it for corrections. RTK, on the other hand, uses the carrier wave to determine the number of cycles between the satellite and the receiver and performs corrections. Without the use of DGPS, it is possible to build a dynamic DGPS with a distributed GPS configuration and with the method proposed in [55]. Although results with decent accuracy were achieved with DGPS and RTK, discontinuity of GPS signals in urban environments and tunnels remains the main issue with these sensors.

GNSS needs to be integrated with other sensors that can compensate for the signal during any possible outage. IMU is a type of sensors that exploit built-in accelerometers and gyroscopes to measure both acceleration and velocity [116]. It then processes this information to estimate the state of the vehicle at a given time with respect to its original position. It is worth mentioning that IMUs suffer from drift error, which results from the accumulation of positioning error during the travel of the vehicle. Hence, an IMU need continuous correction to its estimated position. Despite this, the well-fused output of both GNSS and IMU achieves a state estimate of the vehicle and ensures a continuous localization process.

Various examples of GNSS/IMU sensor fusions exist in the literature. In [117,118,119], the Kalman filter was developed to integrate the outputs of both GPS and IMU. A Kalman filter (KF) consists of two main equations, named the prediction equation, which is based on the system knowledge (evolution matrix and command matrix) obtained from past measurements, and the update equation, which works on updating the knowledge from the current measurements, i.e., an update of the predicted estimation with the Kalman gain and the error between the predicted state vector and the new observation (GPS data). Generally, enormous improvements happen when fusing both sensors using the Kalman filter approach. Nonetheless, it is important to emphasize that the success of using Kalman-filter-based fusion relies on the perfect match between the state–space system model and the measurement model. Additionally, multiple assumptions should be taken into consideration, such as the linearity of the system and the presence of Gaussian distributed data.

Since system dynamics are not always represented with linear equations, an extension named the extended Kalman filter (EKF) was found to handle nonlinear systems. EFK works by linearizing the system’s equations at each time step through the Taylor series and Jacobian matrix, then passing them through the ordinary KF. The main disadvantage of this approach lies in the process of approximation (linearization stage), as it introduces errors that will not be taken into consideration. The unscented Kalman filter (UKF) was next introduced to improve the performance of EKF. Instead of taking one point and approximating it to its linear state, UKF considers a group of weighted points, named sigma points, and uses them for approximation. UKF has achieved better performance in terms of accuracy compared to EKF [120]. Nevertheless, EKF and UKF are mono-model approaches. In order to improve these two well-known approaches, multiple models and multiple hypothesis methods have been developed. Included among these complex approaches are the particle filter approach, the interacting multiple model (IMM) approach [121], and the optimized Kalman particle swarm (OKPS) approach, which is a merge of the particle filter and swarm method [122].

One of the key challenges in estimation methods is the need to have an accurate model of the system. In some cases, it is difficult to provide an accurate model, especially for complex systems that are highly dynamic. Additionally, most sensors are subject to inherent uncertainties, which usually cannot be incorporated in the system model, yielding an inaccurate model [123]. In this context, deep learning is extremely useful, as it allows for end-to-end learning, which eliminates the need for mathematical modeling of each sensor individually.

Based on the current literature, there are few studies on deep learning sensor fusion in localization. An early attempt to deploy artificial intelligence to fuse GNSS and INS is presented in [123], where an input-delayed neural network is utilized to model the errors of the INS based on current and previous data samples. The test results are compared to the conventional Kalman filter approach and they show several improvements in position estimation during GNSS signal outage.

RNN is a powerful tool that can be used with time-series data. It has the ability to save previous data samples through its memory feature. In [124], RNN was used to fuse both GNSS and INS sensors and produce continuous and reliable navigation. Through the recursive network and memory function, RNN uses past position and velocity data of INS to predict the errors in the current measurements. The proposed method showed a 60% improvement when compared to the conventional EKF method and 30% improvement compared to other neural network methods. Similarly, Kim et al. [125] integrated both GNSS and IMU data using long short-term memory (LSTM), a variance of the RNN algorithm. The purpose is to generate a model of the vehicle position estimation without the need to model each sensor analytically. The LSTM network was trained with GNSS absolute position and IMU data, and the predicted position was compared with the reference position obtained from a high-accuracy RTK GPS. While the results of the study aim to validate the use of LSTM as a fusion technique, the study needs to be further enhanced by testing it in real-life complex driving situations.

In a different context, Jiang et al. proposed the use of deep learning algorithms in [126] to model the INS signal noise in order to eliminate it, which improved the navigation system outcomes. In general, different statistical methods or artificial intelligent methods have been used to model the error signal, but all techniques have their own limitations [126]. To overcome those limitations, the RNN algorithm, along with a combination of LSTM and gated recurrent units (GRUs), was used for noise modeling. Due to the training accuracy of LSTM and the convergence efficiency of GRU, significant improvements were reported by the proposed hybrid algorithm.

### 4.2. Visual-Based Localization

Vision sensors are important elements in localization and mapping. Compared to other existing usable sensors, such as LiDAR and radar imaging, cameras are often chosen due to their low cost, availability, and ability to capture useful information (static and persistent primitives in the environment). Visual localization has been an active research area for autonomous vehicles. Visual-based localization includes (1) SLAM, (2) visual odometry (VO), and (3) map-matching-based localization. This section aims to review the contribution of deep learning algorithms in advancing each of the previous methods.

#### 4.2.1. Simultaneous Localization and Mapping (SLAM)

SLAM is an algorithm that combines a set of sensors to build a map of the AV and its surroundings, while simultaneously keeping track of the vehicle’s current position in reference to the built map. Although SLAM algorithms were initially applied in the field of mobile robots, researchers have put a noticeable effort into adjusting the algorithms to suit autonomous vehicle applications. This was done by taking into consideration different key challenges, such as the need for faster processing, the outdoor lighting conditions, and the dynamic road obstacles. It is important to point out that while SLAM mainly relies on vision-based sensors, other sensors such as GPS, LiDAR, and sonar have also been used to implement SLAM algorithms. Surveys on recent SLAM methods have been done by [53,127]. Additionally, some new methods with performance evaluations are available on the KITTI website [128]. At this moment, the latest and best methods reported in [128], which do not use deep learning approaches, are presented in Table 6**.** Different algorithms based on different perception types are compared in Table 6 in terms of their accuracy (translation and rotation error) and the time required to run the algorithm (running time). The performance data shows that “traditional” algorithms generally do well in real-time SLAM implementations. Nonetheless, the continuous progress of SLAM algorithms is still an interesting and active research topic in the computer science and robotics community. It seems that in order to improve “traditional methods”, it is relevant to share static and dynamic spaces. Such an approach has been proposed by [129]. From LiDAR data, this approach shares the dynamic space (detection and tracking of dynamic obstacles) and static space using a belief plot map concept. The interesting aspect of this method is its ability to model and account for large size objects with nonlinear and complex shapes.

Deep learning approaches have shown great improvements in image classification and detection; hence, there is good potential in applying these algorithms to enhance traditional SLAM algorithms. Although the deep learning applications in this field are still not mature, some studies propose replacing parts of classical SLAM blocks with deep learning modules to attain better accuracy, efficiency, reliability, and robustness. These studies include attempts to improve aspects of pose and depth estimations, loop closure detection, and feature descriptors of classical SLAM algorithms.

A very crucial aspect of a reliable SLAM system is its ability to perform well in dynamic environments. Most conventional SLAM algorithms were designed to operate in a static environment; hence, their overall performance, and in particular their accuracy, is dramatically compromised in real-world driving scenarios, where objects are often dynamic and the driving conditions are sometimes unpredictable. Traditionally, the behavior of dynamic objects in SLAM was estimated through filtering or tracking. However, most of these approaches require immense computational power and are impractical in real-time applications. For this purpose, Kaneko et al. [130] utilized deep-learning-based semantic segmentation to exclude feature points that exist in the sky and moving cars. These two categories are segmented and masked, and hence all feature points in the masked area are excluded. Similarly, Xiao et al. [131] used an SSD network as an object detection framework, whereby the output of the network is segmented into static objects and dynamic objects. The latter are considered as outliers and discarded. The proposed method reported higher accuracy compared to the baseline SLAM algorithm. A more generic solution was proposed in [132], where pixel-wise deep semantic segmentation was used to produce semantic labels. A tracking thread will generate feature points, out of which those belonging to moving objects will again be considered as outliers and excluded by an outlier rejection network.

One of the ongoing challenges in SLAM systems is the ability of the sensors to accurately measure the depth of the scene as captured by its vision sensors (stereo vision or optical flow). Although in some cases depth sensors and RGB-D are used, these sensors have shortcomings, such as their inadequate working range and their poor performance under direct sunlight. As an attempt to improve depth estimation, researchers in [82,83] trained a deep learning CNN network to estimate depth using a single monocular camera. Compared to classical monocular SLAM [133], CNN-based SLAM employs learning abilities to learn the absolute scale and eliminate the need for geometric assumptions to correct the scales of detected objects [134]. Another example of CNN-based depth estimation is presented in [135], where a real-time algorithm named DeepFusion is used to reconstruct dense maps by fusing the depth prediction of a stereo camera with the depth and gradient predictions of a CNN network in a probabilistic manner. For further improvement, Lee et al. [136] proposed the addition of a recurrent network to the existing CNN network to account for the spatiotemporal information in the image or video sequence for better depth estimation. In [137], Kuznietsov et al. proposed using a semisupervised deep learning method that can take the advantages of both supervised and unsupervised methods. The proposed technique uses the sparse ground truth data for learning and utilizes CNN for depth prediction.

Another significant module that contributes much to the accuracy of the SLAM system is loop closure. This module checks the previously visited and mapped places and uses the results to reduce the error of the built map. Previously, classical approaches were used to perform detection and classification, such as bag-of-words (BoW), scale-invariant feature transform (SIFT), and speeded-up robust features (SURF) approaches. These approaches use appearance-based methods that are created through handcrafted features and have their own limitations. Deep learning can be highly utilized in the loop closure field, as it has already been proven to be very powerful in image recognition applications. Hou et al. [146] used a pretrained CNN-based descriptor to perform visual loop closure. Merrill et al. [147] also proposed an unsupervised deep autoencoder system for loop closure. The performance of the learning-based method was compared with several hand-crafted techniques under various lighting conditions. CNN has achieved an enhanced performance in the case of major light change and faster extraction speed as well.

Table 7 lists some of the recent deep learning SLAM algorithms. It is worth mentioning that in all of the listed studies, deep learning has been used to replace only a specific module, and the proposed algorithms have generally been built upon a traditional SLAM algorithm. Despite this, deep learning has improved the overall accuracy, and in some cases it has solved critical issues, such as operating in highly dynamic environments. With these continuous improvements, it is conceivable that in the near future there will be an end-to-end deep learning SLAM algorithm with superior accuracy and computational efficiency. Another point that can be observed from Table 7 is the diversity of the testing datasets in the previous studies. Unlike the traditional algorithms presented in Table 6, these algorithms are tested on different datasets; hence, a conclusive comparison of their performance based on the published results is not easy.

#### 4.2.2. Visual Odometry (VO)

Visual odometry (VO) is defined as the process of obtaining the pose of a vehicle by tracking the change of its position from consecutive images over time. A general VO framework consists of camera calibration, image acquisition, feature detection, feature matching, feature tracking, and pose estimation. Traditionally, VO was performed through two main approaches: feature-based approaches, where features such as lines or corners are detected, and appearance-based approaches, in which pixel intensity values are considered instead [150]. Table 8 summarizes some of the recent traditional VO algorithms extracted from the KITTI website. The V-LOAM algorithm [151] is ranked first because of having the smallest reported translational and rotational errors. Despite the outstanding performance of the conventional VO, deep learning has been extensively studied to replace it, as it works as a generic feature extractor and improves the system by eliminating the need to design hard-coded features. Additionally, fine-tuned feature parameters are not required for deep learning; thus, the robustness and reliability of the systems are enhanced, which are otherwise sensitive to changes in the environment. Moreover, deep learning algorithms tend to learn to recover the absolute scales, and hence no prior information on the motion model or camera parameters are needed.

Many research articles attempt to evaluate the performance of deep learning algorithms in pose estimation by comparing their results with traditional feature-based algorithms, such as SURF and ORB. The results from [152] illustrate that a deep-learning-based algorithm performs better than conventional methods. An early study was conducted by the authors in [153], where two CNN networks were used in a supervised fashion with fully connected last layers, which regress the pose of the camera. Several experiments were reported using a combination of known and unknown testing environments. From the results obtained, it was shown that the network performs better with prior knowledge of the environment over unknown environments. However, the results of both cases tend to accumulate errors over time. As a result, it was recommended to add a recurrent network, which will help to alleviate the drift problem.

To demonstrate the advantage of adopting a recurrent neural network for VO, Wang et al. [154] implemented an end-to-end deep recurrent convolutional network that takes sequential RGB images and detects poses. Same authors then extended their study to take uncertainties into account [155]. A CNN network was utilized to extract important feature representations from the image and an RNN network in the form of stacked LSTM was used to process sequential data and model motion dynamics. The proposed method was tested for outdoor driving and the results were comparable to those produced through classical algorithms. However, the performance of the algorithm degraded when under certain conditions, including fast driving or driving in open areas, with fewer features leading to more outliers. One solution is to increase the size of the training dataset for the network to learn to reject the outliers. This requirement triggers a question—is it always possible to increase our testing dataset? The challenge rests in the process of labeling these data. This leads to exploring the field of self-supervised and unsupervised learning.

Unlike supervised learning, an unsupervised learning network does not rely on labeled data or ground truth data for training. Instead, it trains the model by minimizing the photometric error. In [160], Zhou et al. introduced two networks that are jointly trained from unlabeled video frames to predict the depth map and relative camera pose while using view synthesis (i.e., the ability to synthesize a target image by using the depth map and the pose of a nearby image). Li et al. [161] trained the CNN network using stereo images instead of using consecutive monocular images. This approach enabled the network to recover the absolute scale of the scene. In addition to depth and pose estimations, some studies found it essential to incorporate the uncertainty estimation, as the VO problem is considered as a state estimation problem. In [162], the network was trained in an unsupervised manner, but it was further modified to predict the depth and pose, also considering the uncertainty for VO optimization. A summary of the latest deep-learning-based VO studies is listed in Table 9.

### 4.3. Map-Matching-Based Localization

One of the well-known methods of AV localization and mapping is the use of prestored, offline maps, known as “a priori maps”. In this method, a combination of sensors is used to capture the surrounding environment while predriving the area. The sensor outputs are stored to form a detailed map of the driven roads and areas, and later can be compared to current sensor outputs. This approach can achieve centimeter-level localization, as required for AV navigation applications. One of the key challenges in such methods is the need for frequent map updates to match the constantly changing urban and driving environments.

One of the vast emerging technologies in the field of maps is the building process of HD maps. HD maps provide very accurate lane-by-lane information, enabling vehicles to precisely localize themselves with respect to those maps. Several leading companies such as HERE rely on using the latest LiDAR technology to capture 3D point cloud data of different elements on the road, such as lane markings, road curvatures, road obstacles, and road signs. At the same time, they accommodate real-time changes by updating those maps [165] continuously.

In the last decade, localization based on map matching has received significant consideration. In the literature, several different methods and algorithms exist to achieve accurate vehicle localization. Traditional registration methods such as iterative closest point (ICP), Monte Carlo localization, and normal distribution transform (NDT) have reported satisfactory results. Nevertheless, these algorithms are highly dependent on manual calibration, postprocessing fine-tuning, and handcrafted features for matching.

Employing deep learning algorithms for localization is still an open research topic, even though these algorithms have proven their effectiveness in performing detection, classification, and learning semantics. Some recent studies demonstrate promising results, such as the one presented in [166], which aims to localize vehicle position using LiDAR measurements and prestored point cloud map. The proposed method consists of (i) keypoint extraction and feature descriptors, (ii) a 3D CNN network that takes the cost volume and regularizes it in order to find the matching cost between each captured key point and its equivalent location on the map, and (iii) an RNN module that is used to learn historical relations between sequential frames and perform temporal smoothness for smoother trajectory predictions.

In an attempt to improve the accuracy of localization, in [167] Vaquero et al. suggested improving the quality of the prebuilt map first. They proposed segmentation of the dynamic moving objects in the map, such as other vehicles and pedestrians, in order to obtain a map that is valid for use for a longer period. For this, the LiDAR front view and birds eye viewpoint cloud are processed by dual deep CNN networks to perform segmentation for both views and then filter out all the movable objects.

## 5. Conclusions and Future Research Recommendations

The field of autonomous vehicles and self-driving cars is vast, as it involves a great variety of subjects ranging from electronics, sensors, and hardware to control and decision-making algorithms, as well as all the social and economic aspects. For this reason, the research opportunities in this field are endless and have growing potential for future expansion. Prospective AV research areas related to technical aspects can cover more advanced sensor technologies, algorithm enhancement, data collection and storage, communication security, and overall performance improvements. In addition, research domains can be extended to cover nontechnical topics, such as the level of societal acceptance of autonomous driving, environmental effects, changes to urban design, and economic benefits.

In this study, we surveyed and critiqued work on perception, localization, and mapping tasks of autonomous vehicles, particularly those empowered by deep learning algorithms that can take advantage of data-driven knowledge discovery rather than physics-based models. As related to the scope of the study, this section aims to summarize the potential research areas that will possibly improve and enrich the field of autonomous vehicles. The recommendations will focus on both environmental perception, localization and mapping, and how to further utilize deep learning algorithms to improve the performance of sensor fusion networks.

### 5.1. Harsh Weather Conditions

One of the remaining challenges of self-driving cars is their compromised maneuverability and performance in bad weather conditions, such as rain, snow, dust storms, or fog, which can compromise vision and range measurements (degradation of the visibility distance). In such conditions, the performance of most current active and passive sensors is significantly compromised, which in turn leads to erroneous and even misleading outputs. The consequence of a partial or complete sensor failure can be catastrophic for autonomous vehicles and their surroundings. A possible measure to alleviate this problem is to evaluate the risk of failure early in the process based on learned experiences and historical data using deep learning algorithms and to allow the driver to interrupt or completely disengage the autonomous system. Approaching such an issue could go through two main paths. The first path would be to utilize already existing sensors that have complementary outputs and enhance the fusion algorithm through deep learning approaches [168]. The second path would be to invest in the sensor hardware technology, as seen in short-wave gated camera and short-wave infrared LiDAR approaches [169]. Both paths have room for further development and enhancement.

### 5.2. Landmark Map-Matching

Improvement of localization and mapping is an ongoing research topic in the field of AV systems. It is vital to achieving a sub-decimeter accuracy level to avoid collisions and navigate a vehicle safely. One of the recently emerging techniques is to improve localization by detecting repetitive and distinct landmarks, such as light poles, traffic signs, or road markings, and compare their perceived location with an a priori offline map. Most of the previous work relies on traditional fusion algorithms with inefficient detection algorithms [170,171,172]. Replacing those methods with deep learning algorithms will accelerate learning if such landmarks and their possible variations, without the need to define them explicitly. The generalization ability of deep learning methods will enhance the reliability of the landmark matching, as its efficiency has already been proven in many related fields, such as object recognition and detection. An important example is the emergence of 3D computer vision and 3D image understanding, which refer to the analysis and recognition of the objects using volumetric images and point clouds [11,173,174,175]. Benefiting from both visual and geometrical information, 3D or shape-based computer vision methods can be significantly more useful than 2D or image-based methods in landmark recognition and matching. The superiority of 3D computer vision methods is because volumetric images contain more information and features of the objects and are less affected by camouflage, disguise, lighting conditions, image quality, and noise. However, three-dimensional analyses of volumetric images are also more complex, and hence more prone to error, if not treated properly. Thus, the implementation of the 3D computer vision paradigm in real-world settings imposes additional challenges that need to be addressed before it becomes a practical and reliable solution for AV applications.

### 5.3. Deep Learning Algorithms for Localization

Undoubtedly, it can be concluded that deep learning algorithms, in particular CNN, have been heavily applied to perform environment perception. CNN is able to learn features automatically and is very powerful in image-related tasks; hence, it is the ultimate choice for perception, where the majority of the efforts include image recognition and classification. In contrast, applying deep learning to localization has not drawn the same attention or reached the same level of maturity. Thus, there is great potential to apply RNN algorithms to tackle the sequential localization data and improve it further.

Deep learning has been used to replace certain modules of the traditional SLAM algorithms, and so far has improved the performance of localization and mapping to a certain extent. In the future, learning algorithms may offer an end-to-end deep learning SLAM system that can avoid feature modeling and data association, and consequently reduce errors and uncertainties associated with unmodeled dynamics and imperfect modelling. Moreover, similar to the VO end-to-end systems, the SLAM algorithms developed in this fashion will maintain a unified benchmark, making it possible to compare the performance of different approaches.

### 5.4. Issues to Solve: Cybersecurity, Reliability, and Repeatability

While deep learning approaches have dramatically improved different AV perception and localization modules, it is important to stress that these approaches require large datasets that are generated over an extended period of time. The outcomes of these approaches depend on the quality and comprehensiveness of the training datasets, and hence the results could vary in terms of relevance and reliability. Merging classical model-based methods and deep learning approaches can improve the robustness and reliability of the existing methods.

One important concern regarding the certification and homologation of the perception and localization of deep learning-based approaches is to guarantee the maintenance of their high level of efficiency. On the other hand, some recent experiments have revealed the sensitivity of the data-driven approaches to small disturbances and interferences in the sensor data. In [176], for instance, the author proposed adversarial physical conditions, which compromised object recognition and subsequently misled the whole system. AdvHat introduced in [177] is an interesting adversarial attack method that attacks face ID systems. This method can easily breach the best public face ID model. The same approach may be used to attack road perception functions and cause huge damage to the AV system. Additionally, [178,179] introduced an overview that illustrates how deep learning methods can be deceived and breached. Nevertheless, other researchers have demonstrated the robustness of deep learning algorithms against computation failures [180].

## Figures and Tables

**Table 1:** Summary of AV applications, limitations of sensors, and advantages of sensor fusion.

Study	AV Application	Fused Sensors	Limitations without Fusion	Fusion Advantages
[34,35,36]	Pedestrian Detection	Vision and LiDAR	Sensitive to illumination quality; Night vision difficulties by vision camera only Low resolution of LiDAR 3D scene reconstruction when used alone.	Ability to measure depth and range, with less computational power; Improvements in extreme weather conditions (fog and rain)
[37,38,39,40,41,42]	Pedestrian Detection	Vision and Infrared	Night vision difficulties with vision camera only; Thermal cameras lose fine details of objects due to their limited resolution.	Robustness to lighting effects and nighttime detection; Infrared camera provides distinct silhouettes of objects; Ability to operate in bad weather conditions.
[43,44,45,46]	Road Detection	Vision and LiDAR	Illumination and lighting conditions; High computational cost for vision depth measurements; Limited resolution and range measurements by LiDAR; Sparse and unorganized point cloud LiDAR data	Road scene geometry measurements (depth) while maintaining rich color information; Calibration of scattered LiDAR point cloud with the image
[47]	Road Detection	Vision and Polarization camera	Sensitive to lighting conditions; Lack of color information	Polarized images enhance scene understanding, especially with reflective surfaces.
[48,49,50]	Vehicle Detection Lane Detection	Vision and Radar	Low resolution of radar. Camera needs special lenses, arrangements, and heavy computation to measure distance.	Measure distance accurately; Performs well in bad weather conditions; Camera is well suited for lane detection applications
[51]	Visual Odometry	2D Laser scanner and Vision	2D scanners can miss detection of objects in complex environments; 2D images are insufficient for capturing all the features of the 3D world.	Fusion of vision and 2D scanners can replace the need for 3D LiDAR, and hence reduce price and computation load.
[52,53]	SLAM	Vision and Inertial Measurement Unit	Illumination and lighting conditions by the camera; Camera suffers blur due to fast movement; Drifting error for IMU	Improved accuracy with less computational load; Robustness against vision noise, and corrective for IMU drifts.
[54]	Navigation	GPS and INS	GPS outage in denied and canyon areas; Drift in INS readings	Continuous navigation; Correction in INS readings
[32,55]	Ego Positioning	Map, vision, GPS, INS	GPS outage; INS drifts; HD map accuracy; Visibility of road markings	Accurate lateral positioning through road marking detection and HD map matching.

**Table 2:** A comparison between traditional sensor fusion algorithms, their advantages, disadvantages, applications, and fusion level.

Algorithm	Characteristics	Advantages	Disadvantages	Applications Areas	Level of Fusion
Statistical Methods	Utilized to enhance data imputation using a statistical model to model the sensory information [64,67]	Can handle unknown correlations; Tolerant [68,69]	Limited to linear estimators; Computation complexity is high [65]	Estimation	Low [70]
Probabilistic Methods	Based on probability representation for the sensory information [64]	Uncertainty in the provided information is handled. handles nonlinear systems (particle filter, UKF, …)	Requires prior knowledge of systems model and data	Estimation/Classification	Low→Medium [70]
Knowledge-based Theory Methods	Utilizes computational intelligence approaches inspired by human intelligence mechanisms. [71]	Handles Uncertainty and imprecision; Ability to handle complex nonlinear systems [72]	Depends on the expertise knowledge and extraction of knowledge	Classification/Decision	Medium→High [70]
Evidence Reasoning Methods	Depends on the Dempster combination mechanism to implement the model [71]	Uncertainty degree is assigned to the provided information. Identification of conflicting situation. Modeling of complex assumption	High computation complexity. Require assumption of evidence.	Decision	High [70]
Interval Analysis theory	Shares the operating space in intervals [73]. Constraint satisfaction problem [74,75]	Guaranty integrity. Ability to handle complex nonlinear systems	Discretization of the operating space. High computation complexity.	Estimation	Low

**Table 3:** A summary of different deep learning algorithms, their main properties, and applications.

DL Algorithm	Description	Applications
Convolutional Neural Network (CNN)	A feedforward network with convolution layers and pooling layers. CNN is very powerful in finding the relationship among image pixels.	Computer Vision [82,83,84]; Speech Recognition [85]
Recurrent Neural Network (RNN)	A class of feedback networks that uses previous output samples to predict new data sample. RNN deals with sequential data; both the input and output can be a sequence of data.	Image Caption [86]; Data Forecasting [87]; Natural Language Processing [88]
Deep Belief Net (DBN)	Multilayer generative energy-based model with a visible input layer and multiple hidden layers. DBN assigns probabilistic values to its model parameters.	Collaborative Filtering [89]; Handwritten Character Recognition [90]; acoustic modeling [91]
Autoencoders (AE)	A class of neural network that tends to learn the representation of data in an unsupervised manner. AE consists of an encoder and decoder, and it can be trained through minimizing the differences between the input and output.	Dimensionality Reduction [92]; Image Retrieval [93]; Data Denoising [94]

**Table 4:** Comparison of the training time and testing time of different region-based detection algorithms and improvements of each algorithm compared to R-CNN.

	R-CNN	SPP-Net	Fast R-CNN	Faster R-CNN
Training time (In hours)	84	25	9.5	NA
Speedup with respect to R-CNN	1×	3.4×	8.8×	NA
Testing rate (Seconds/Image)	47	2.3	0.3	0.2
Speedup with respect to R-CNN	1×	20×	146×	235×

**Table 5:** Comparison of localization and mapping techniques in terms of the accuracy, cost, computational load, source of external effects, and the storage size of data.

Method	Accuracy	Cost	Computational Load	External Effect	Data Size
GPS/IMU	Low	Medium	Low	Signal outage	Low
GPS/INS/LiDAR/Camera	High	Medium	Medium	Map accuracy	High
SLAM	High	Low	High	Illumination	High
Visual Odometry	Medium	Low	High	Illumination	High
Map-Based Matching	Very High	Medium	Very high	Map change	Very High

**Table 6:** SLAM algorithms based on non-deep-learning approaches, as reported on the KITTI website.

Date	Reference	Method	Translation	Rotation	Runtime	Sensor
2017	[138]	SOFT-SLAM 2	**0.65%**	**0.0014**	0.1 s	Stereo
2018	[139]	LG-SLAM	0.82%	0.0020	0.2 s	Stereo
2017	[140]	ORB-SLAM2	1.15%	0.0027	**0.06 s**	Stereo
2015	[141]	S-LSD-SLAM	1.20%	0.0033	0.07 s	Stereo
2018	[142]	IMLS-SLAM	0.69%	0.0018	1.25 s	LIDAR
2018	[143]	MC2SLAM	**0.69%**	**0.0016**	**0.1 s**	LIDAR
2018	[144]	CPFG-slam	0.87%	0.0025	0.03 s	LIDAR
2018	[145]	SuMa	1.39%	0.0034	0.1 s	LIDAR

Translation: relative translation error in percentage; rotation: relative rotation error in degrees per 100 m. Data in bold represents highest performance.

**Table 7:** Summary of recent deep-learning-based SLAM algorithms.

Year	Reference	Contribution of Deep Learning	Description	Architecture	Testing Datasets	Runtime
2018	[130]	Semantic Segmentation	Semantic segmentation produces a mask and the feature points on the mask are excluded.	DeepLab V2	CARLA	-
2019	[148]	Feature Descriptors	Replace handcrafted descriptors with learned feature descriptors.	TFeat	EuRoC/TUM	90 ms
2018	[132]	Semantic Segmentation	Semantic segmentation reduces the effect of dynamic objects and is used to build a dense map.	SegNet	TUM/Real Environment	76.5 ms
2019	[131]	Semantic Segmentation	SSD Network is used to detect dynamic objects. The selection tracking algorithm is used to eliminate dynamic objects and a missed detection compensation algorithm is used for improvements.	SSD	TUM/KITTI	45 ms
2018	[149]	Pose Estimation	End-to-end trained model that consist of a local pose estimation model, pose selection module, and graph optimization process.	FlowNet DTC	Viz-Doom simulated maze	-
2018	[147]	Loop Closure	Compact unsupervised loop closure algorithm that is based on convolutional autoencoders.	Autoencoders	KITTI	-
2019	[135]	Depth Estimation	Real time algorithm that is able to reconstruct dense depth maps from RGB images.	U-Net	ICL-NUIMTUM RGB	94 ms
2020	[136]	Depth Estimation	A recurrent CNN network that is used to process spatial and temporal information for map depth estimation.	Convolutional GRU (U-Net)	KITTI	80 ms

**Table 8:** Summary of recent VO approaches based on non-deep-learning approaches, as reported on the KITTI website.

Date	Reference	Method	Translation	Rotation	Runtime	Sensors
2015	[151]	V-LOAM	**0.54%**	**0.0013**	**0.1 s**	MC + LIDAR
2019	[143]	MC2SLAM	0.69%	0.0016	0.1 s	IMU + LIDAR
2018	[156]	LIMO2_GP	0.84%	0.0022	0.2 s	MC + LIDAR
2017	[157]	GDVO	0.86%	0.0031	0.09 s	SC
2018	[156]	LIMO	0.93%	0.0026	0.2 s	MC + LIDAR
2018	[156]	LiViOdo	1.22%	0.0042	0.5 s	MC + LIDAR
2019	[158]	SALO	1.37%	0.0051	0.6 s	LIDAR
2019	[159]	KLTVO	2.86%	0.0044	0.1 s	SC

Translation: relative translation error in percentage; rotation: relative rotation error in degrees per 100 m. Data in bold represents highest performance.

**Table 9:** Recent VO algorithms based on deep learning approaches.

Year	Reference	Description	Architecture	Testing Datasets	Learning Model
2017	[155]	End-to-end algorithm for finding poses directly from RGB images using deep recurrent convolutional neural networks.	CNN-LSTM	KITTI	Supervised
2019	[163]	Encode-regress network that produces 6-Degree of Freedom (DoF) poses without the need of depth maps.	ERNet	KITTI	Semi-Supervised
2016	[153]	Two parallel CNN networks are connected at the end by fully connected layers to generate the required pose.	AlexNet	KITTI	Supervised
2017	[160]	An end-to-end algorithm that uses single-view depth and multi-view pose for camera depth and motion estimation.	DispNet	KITTI	Unsupervised
2017	[152]	An approach that generates a 7-dimensional relative camera pose orientation and position vector.	AlexNet with SPP	DTU	Supervised
2018	[161]	Pose and dense depth map estimation with an absolute scale. This generates 6 DoF poses from unlabeled stereo images.	VGG-16 and Encoder-Decoder	KITTI	Unsupervised
2020	[162]	The algorithm uses deep networks for depth, pose, and uncertainty estimation of monocular odometry.	U-Net (DepthNet and PoseNet)	KITTI EuROC MAV	Unsupervised
2018	[164]	A global pose regression and relative pose estimation framework. The network takes two monocular frames and regresses the 6 DoF poses with inter-task correlation.	ResNet-50	Microsoft 7-Scenes Cambridge Landmarks	Supervised
